# MICT ameliorates hypertensive nephropathy by inhibiting TLR4/NF-κB pathway and down-regulating NLRC4 inflammasome

**DOI:** 10.1371/journal.pone.0306137

**Published:** 2024-07-25

**Authors:** Wenyu Dong, Minghao Luo, Yun Li, Xinhua Chen, Lingang Li, Qing Chang

**Affiliations:** 1 The Affiliated Rehabilitation Hospital, Chongqing Medical University, Chongqing, P. R. China; 2 Department of Cardiology, The First Affiliated Hospital of Chongqing Medical University, Chongqing, P. R. China; 3 The College of Exercise Medicine, Chongqing Medical University, Chongqing, P. R. China; King Faisal University College of Veterinary Medicine and Animal Resources, SAUDI ARABIA

## Abstract

**Background:**

Hypertensive nephropathy (HN) is one of the main causes of end-stage renal disease (ESRD), leading to serious morbidity and mortality in hypertensive patients. However, existing treatment for hypertensive nephropathy are still very limited. It has been demonstrated that aerobic exercise has beneficial effects on the treatment of hypertension. However, the underlying mechanisms of exercise in HN remain unclear.

**Methods:**

The spontaneously hypertensive rats (SHR) were trained for 8 weeks on a treadmill with different exercise prescriptions. We detected the effects of moderate intensity continuous training (MICT) and high intensity interval training (HIIT) on inflammatory response, renal function, and renal fibrosis in SHR. We further investigated the relationship between TLR4 and the NLRC4 inflammasome *in vitro* HN model.

**Results:**

MICT improved renal fibrosis and renal injury, attenuating the inflammatory response by inhibiting TLR4/NF-κB pathway and the activation of NLRC4 inflammasome. However, these changes were not observed in the HIIT group. Additionally, repression of TLR4/NF-κB pathway by TAK-242 inhibited activation of NLRC4 inflammasome and alleviated the fibrosis in Ang II-induced HK-2 cells.

**Conclusion:**

MICT ameliorated renal damage, inflammatory response, and renal fibrosis via repressing TLR4/NF-κB pathway and the activation of NLRC4 inflammasome. This study might provide new references for exercise prescriptions of hypertension.

## 1 Introduction

Hypertension is one of the major risk factors for vascular and cerebrovascular disease, its incidence is continuously increasing worldwide [1]. Persistently elevated blood pressure can damage renal blood vessels, leading to chronic hypertensive kidney damage, which is also the main cause of end-stage renal disease [2]. Hypertensive nephropathy (HN) refers to renal arteriosclerosis, thickening of the renal artery wall, and narrowing of the lumen caused by high blood pressure, inducing substantial ischemic damage of kidney and leading to renal function failure ultimately [3]. However, the current treatment methods for HN are still very limited, mainly to control blood pressure and delay progressive damage to renal parenchyma [4].

Substantial studies have demonstrated that chronic inflammation is involved in the pathogenesis of HN [5–7]. During chronic inflammation, both tissue remodeling and repair processes appear simultaneously [8]. Release of pro-inflammatory cytokines and infiltration of inflammatory cells trigger for the fibrosis process, leading to renal fibrosis and failure [9]. The body’s first line of defensing against the invasive microorganisms is the innate immune system [10]. The innate immune system contains several pattern recognition receptors (PRRs), including membrane-bound Toll-like receptors (TLRs) and nucleotide-binding domain leucine-rich oligomerization domain (NOD)-like receptors (NLRs) [11]. These receptors recognize pathogen associated molecular patterns (PAMPs) and danger associated molecular patterns (DAMPs) in both extracellular and intracellular [12]. Toll-like receptor 4 (TLR4) is one of the core members of the TLRs family, plays an important role in innate immune response [13]. Activated NLRs and apoptotic associated speckle-like protein (ASC) interact each other to activate Caspase-1 to generate inflammasomes [14]. Inflammasomes are large molecular complexes activated during cell infection, which can induce the maturation of pro-inflammatory cytokines including interleukin-1β to participate in fibrosis development [15–17]. There are four main members of the inflammasomes, including NOD-like receptor protein 1 (NLRP1), NOD-like receptor protein 3 (NLRP3), NOD-like receptor C4 (NLRC4), and absent in melanoma 2 (AIM2) [18]. NLRP3 inflammasome has been shown to participate in the pathogenesis of hypertensive nephropathy [19–21]. However, there is currently limited research on NLRC4 inflammasome, and the role of NLRC4 in fibrosis remain to be studied [22].

Increasing evidence indicate that exercise is an effective treatment for chronic cardiovascular diseases such as hypertension [23–25]. Exercise can delay renal failure by inhibiting renal fibrosis, its underlying mechanism still needs to be elucidated [26]. Moderate intensity continuous training (MICT) has always been used to prevent hypertension due to its effectiveness and safety [27]. High intensity interval training (HIIT) is a type of physical exercise that alternates relatively intense activity (85–100% of VO_2_ peak) and brief rest or low-intensity activity to allow physical recovery [28]. In recent years, HIIT has been proposed as a time-efficient and superior treatment to MICT in reducing blood pressure (BP) in individuals with hypertension [29]. The objectives in this work were to determine the role of NLRC4 in hypertensive nephropathy and compare the effects of MICT and HIIT on NLRC4.

## 2 Materials and methods

### 2.1 Animals and experimental protocols

Specific-pathogen-free, eight-week-old male SHRs (n = 18) and Wistar Kyoto Rats (WKYs, n = 6) were obtained from Vital River Laboratory Animal Technology Co. Ltd. (Beijing, China). All of the rats were randomly divided into four groups: The WKY sedentary group (WKY, n = 6), the SHR sedentary group (SHR-S, n = 6), the moderate-intensity continuous exercise group (SHR-M, n = 6), and the high-intensity interval exercise group (SHR-H, n = 6). All rats were maintained under a controlled temperature room (22 ± 2°C) on a 12h/12h light/dark cycle, and were available to chow and tap water *ad libitum*. To alleviate the suffering of rats, we anesthetized them before taking blood and kidney samples. After anesthetized rats with 3% pentobarbital (60 mg/kg) via intraperitoneal injection, rat blood and bilateral kidneys were collected. All rats were sacrificed with CO_2_ suffocation. The animal experiments were approved by the Ethics Committee of Chongqing Medical University (Approval no. 2022136) and performed in accordance with the Chinese Animal Protection Laws and Institutional Guidelines.

### 2.2 Exercise training

A treadmill specifically designed for animal exercise was used throughout the study. Rat exercise protocol was based on guidelines for animal exercise training protocols for cardiovascular studies and our previous research [30–32]. During the first week, adaptive training was conducted with the SHR-M and SHR-H groups, which were trained to run at 10 m/min (0° slope) for 60 minutes. The SHR-M group ran at a speed of 18m/min and approximately 50% of maximum oxygen uptake (VO_2max_) then gradually accelerated at a speed of 1 m/min in every 3 weeks up to 20 m/min (0° slope). Similarly, the SHR-H group ran at a speed of 45m/min and approximately 90% of VO_2max_ then gradually accelerated at a speed of 1 m/min in every 3 weeks up to 47 m/min (0° slope). In each exercise training, the rats underwent a two-minute break every 30 seconds of running. The SHR-M and SHR-H groups were performed a 60 min exercise training every day for 5 days a week for 8 weeks in total. Whereas, WKY group and SHR-S group as control groups were raised quietly for 8 weeks. After the exercise training of 8 weeks, the rats were maintained in the metabolic cages for 24 h to collect 24-hour urine for quantitative urinary protein testing. Serum and kidney were kept at -80°C for future research.

### 2.3 Measurements of weight, heart rate and blood pressure

The weight of rats was measured using an animal scale every week. Systolic blood pressure (SBP), diastolic blood pressure (DBP), mean arterial pressure (MAP) and heart rate (HR) were measured in conscious rats by applying the indirect tail cuff method (BP98A, Softron, Tokyo, Japan).

### 2.4 Renal histopathological analysis

The kidney tissue was fixed in 4% formaldehyde for 24 hours, embedded in paraffin and cut into 5-μm-thick sections for pathological analysis. After being dewaxed and hydrated, Hematoxylin—eosin staining was conducted to observe pathological injury in renal tissue following the manufacturer’s instructions (Solarbio, Beijing, China). Masson trichrome staining was performed to evaluate the degree of renal tissue fibrosis following the manufacturer’s instructions (Solarbio, Beijing, China).

### 2.5 Cell culture

Human renal proximal tubule epithelial cells (HK-2) were purchased from American Type Culture Collection (Manassas, USA). Cells were cultured in Dulbecco’s modified Eagle’s medium supplemented with 10% fetal bovine serum (Gibco, Australia) and 1% penicillin-streptomycin (Beyotime, Shanghai, China) at 37°C with 5% CO_2_.

### 2.6 Cell viability assay

Cell viability was assessed by the Cell Counting Kit-8 assay (CCK-8, Beyotime, Shanghai, China). The HK-2 cells (5 × 10^3^ cells/well) were seeded in 96-well plates and serum starvation for 24 h, then treated with angiotensin II (Ang-II; MedChemExpress) of 1, 5, 10, 20, 40 μM for 24 h to determine the optimal concentration of Ang-II for inducing cell injury. After discarding the medium, 10% CCK-8 solution was then added to each well and the cells were cultured in dark for 1 h. The absorbance was measured at 450 nm on an automatic microplate reader (Thermo Scientific, USA).

### 2.7 Western blotting

The kidney tissues and HK-2 cells were lysed with a RIPA lysis buffer (Epizyme, Shanghai, China) containing 1% protease and phosphatase inhibitors (Beyotime, Shanghai, China), followed by sonication of 15 seconds. The homogenates were centrifuged at 12, 000 rpm for 15 min at 4°C, and then, the supernatants were collected and mixed with 5x loading buffer in a ratio of 4:1 to prepare protein samples. Total proteins (30 μg) were separated by 10% and 12.5% sodium dodecyl sulfate-polyacrylamide gels (Epizyme, Shanghai, China) and transferred to polyvinylidene difluoride membranes. The membranes were blocked in 5% non-fat milk at room temperature for 2 h and then were incubated in primary antibodies at 4°C overnight. The primary antibodies used were anti-Collagen I (1:1000; Zenbio), anti-Collagen Ш (1:1000; Huabio), TGF-β1 (1:1000; Zenbio), α-SMA (1:1000; Zenbio), anti-TLR4 (1:1000; Proteintech), anti-NF-κB P65 (1:1000; Zenbio), anti-P-P65 (1:500; Zenbio), anti-IкBα (1:1000; Zenbio), and anti-p-IкBα (1:500; Zenbio), anti-NLRC4 (1:1000; AiFang), anti-Caspase-1 (1:1000; Zenbio), anti-ASC (1:1000; Zenbio), anti-IL-1β (1:500; Huabio), anti-GAPDH (1:50000; Proteintech). Membranes were washed in TBST (0.01% Tween) 3 times for 30 minutes then incubated with HRP-conjugated secondary antibody for 1 h at room temperature. Using chemiluminescence substrate kit, the signals were visualized with ChemiDoc Imaging System (Bio-Rad Laboratories, United States). Band gray values were analyzed by ImageJ software, GAPDH were used as the internal control.

### 2.8 Biochemical analysis

To evaluate renal function of rats, blood urea nitrogen, serum creatinine and urine protein were detected using the corresponding kits obtained from Nanjing Jiancheng Bioengineering Institute (Nanjing, China). All procedures followed the manufacturer’s instructions respectively.

### 2.9 Immunohistochemical staining

The kidney sections were kept in a 60°C oven for 2 h. Paraffin sections were dewaxed in xylene for 20 minutes, then rehydrated in gradient ethanol (100%, 95%, 85%, and 75%) for 20 minutes, and washed in distilled water for 10 minutes before staining procedures. Following sections were placed in the antigen repair solution and boiled in the microwave oven for antigen repair, the sections were treated with 3% H_2_O_2_ at room temperature for 30 minutes, blocked with 5% goat serum for 30 min, and incubated with the anti-TLR4 at 4°C overnight. Afterwards, sections were incubated with matched secondary antibody for 1 h at room temperature. After being stained with 3, 3-diaminobenzidine (DAB), the sections were counterstained with hematoxylin. Followed by dehydrating and drying, the sections were fixed with neutral gum. Slices were visualized and photographed using a microscope (Olympus, Tokyo, Japan).

### 2.10 Enzyme-linked immunosorbent assay

The serum of rats was tested for interleukin-1β (IL-1β), interleukin-6 (IL-6) and tumor necrosis factor-α (TNF-α) by using enzyme-linked immunosorbent assay (ELISA) kits (MULTISCIENCES, Hangzhou, China), according to the corresponding manufacturer’s instructions. Then, the spectrophotometer (Thermo Fisher Scientific, USA) was used to measure absorbance at a wavelength of 450nm. Finally, the expression levels of the pro-inflammatory cytokines were calculated using a standard curve.

### 2.11 Statistical analysis

Data are expressed as means ± standard error of mean (SEM). Data between two groups were analyzed with an unpaired Student’s t-test. Data among different groups were analyzed with one-way ANOVA. All analyses were performed by the GraphPad Prism 9.0 software. The difference was considered statistically significant if *p* < 0.05.

## 3 Results

### 3.1 Basic data

**Fig 1:**
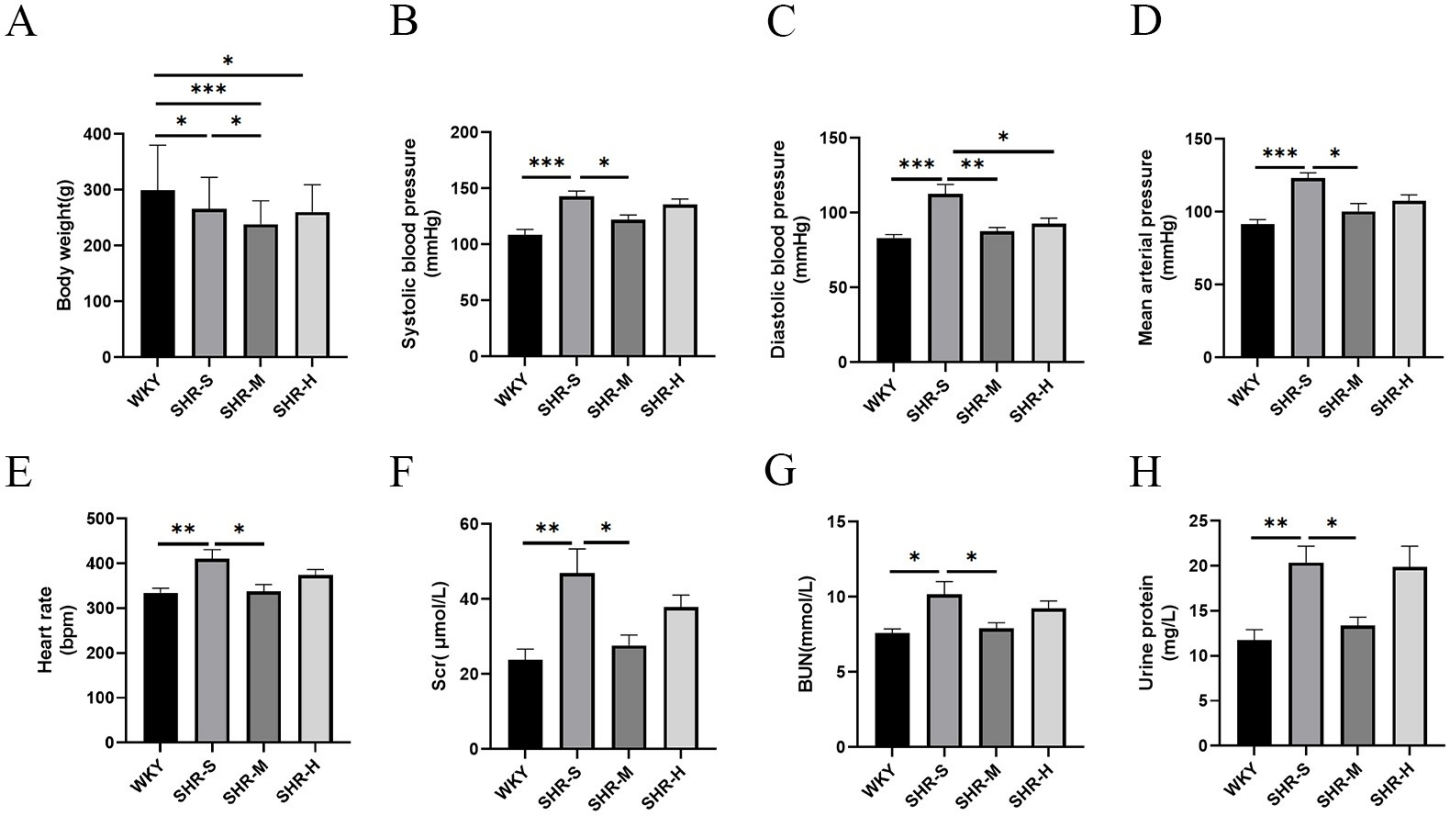
Effects of MICT and HIIT on body weight, blood pressure, heart rate and renal function. SHR-M group and SHR-H group performed exercise training with different exercise prescriptions for 8 weeks. (A) The weight of rats was measured every week. (B) Systolic blood pressure, (C) Diastolic blood pressure, (D) Mean arterial pressure and (E) Heart rate, were measured after 8-week exercise training. (F) Serum creatinine (Scr), (G) Blood urea nitrogen (BUN), and (H) Urine protein were measured to evaluate the renal function. *p< 0.05, **p< 0.01, ***p< 0.001.

To examine the effects of exercise training on the weight of rats, we measured the weight of rats after weekly exercise training. The weight of SHR-S, SHR-M and SHR-H groups was statistically decreased compared with the WKY group. The SHR-M group had significantly lower weight than that in the SHR-S group. The weight of SHR-H group was similar to SHR-S group. After 8-week exercise training, the final HR, SBP, DBP, and MAP of SHR-M group were lower than those in the SHR-S group. However, a decrease in DBP was observed in the SHR-H group than that in SHR-S group (Fig 1A–1E).

We measured the levels of BUN, Scr and urine protein to evaluate the severity of kidney injury. The results suggested that SHR-S group had higher levels of Scr, BUN, and urine protein than the WKY group. SHR-M group, compared to SHR-S group, showed significant decreased in BUN, Scr and urine protein. However, HIIT had no significant effects on the levels of Scr, BUN, and urine protein (Fig 1F–1H).

### 3.2 Effects of exercise training on renal pathological damage

**Fig 2:**
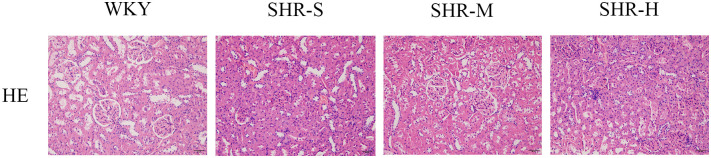
Effects of MICT and HIIT on renal pathological changes. H&E staining was used to observe changes of pathology of kidney tissue (magnification, 200×). WKY group: there was a normal kidney tissue with normal morphology of renal glomeruli and tubules. SHR-S group: there was an abnormal kidney tissue with abnormal glomerular morphology, tubular vacuoles, and renal capsule dilation. SHR-M group showed no obvious pathological manifestations. In addition to pathological manifestations of abnormal glomeruli and tubules, the SHR-H group also showed infiltration of inflammatory cells.

We performed hematoxylin-eosin staining (H&E staining) to evaluate the effects of exercise training on renal pathological damage in SHR. H&E staining showed that SHR-S group exhibited abnormal pathological manifestations including abnormal glomerular morphology, tubular vacuoles, and renal capsule dilation, which was alleviated in the SHR-M group. But HIIT did not improve the abnormal renal pathological manifestations (Fig 2).

### 3.3 Effects of exercise training on renal fibrosis

**Fig 3:**
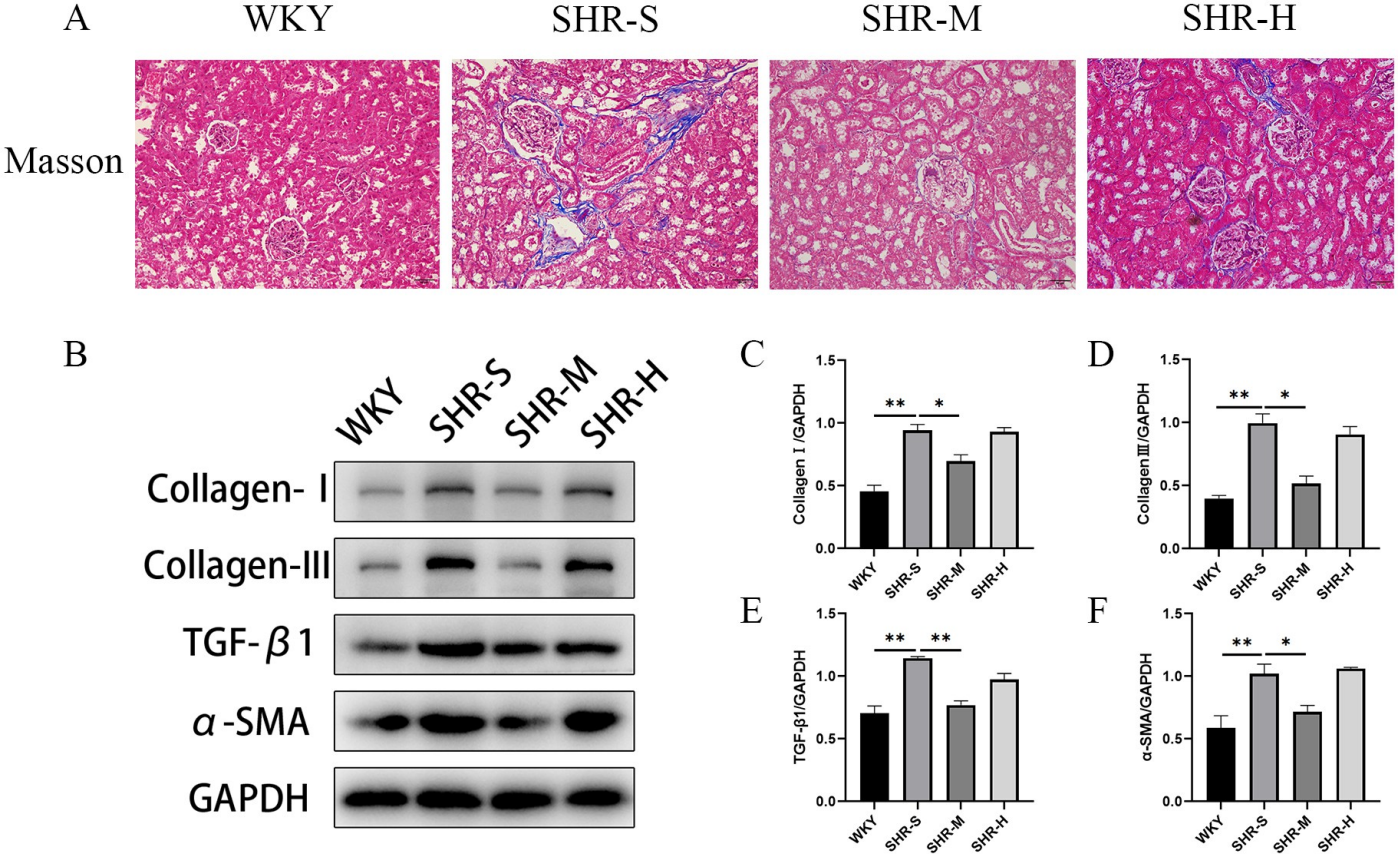
Effects of MICT and HIIT on renal fibrosis in SHR. (A) Masson trichrome staining was performed to evaluate the degree of renal fibrosis. Blue staining represents renal fibrosis. (B) Western blot was used to measure the levels of (C) collagen I, (D) collagen III, (E) TGF-β1 and (F) α-SMA in WKY group, SHR-S group, SHR-M group, SHR-H group. Data are presented as means ± SEM. N = 3 in each group. *p< 0.05, **p< 0.01.

We performed Masson trichrome staining and Western blot to investigate the effects of exercise training on renal fibrosis in SHR. The Masson trichrome staining results suggested that the fibrotic area of renal cortices in the SHR-S group was much larger than the WKY group. Furthermore, a significant decrease was observed in the SHR-M compared with the SHR-S group, whereas there was no significant difference was observed between SHR-S group and the SHR-H group (Fig 3A).

Collagen I and collagen III are the main elements of extracellular matrix (ECM), and their expression levels can reflect the deposition of extracellular matrix [33]. TGF-β1-mediated signaling has long been identified as a driver of collagen accumulation and tissue fibrosis [34]. α-SMA is regarded as an indicator of mature myofibroblast and reflects the severity of kidney fibrosis [35]. The results suggested that the expression levels of collagen I, collagen III, TGF-β1 and α-SMA increased markedly in SHR-S group in comparison to WKY group. Moreover, the expression levels of fibrosis-related proteins were significantly decreased in SHR-M group compared with the SHR-S group. We found no distinct differences between SHR-S group and SHR-H group in the expression of fibrosis-related proteins (Fig 3B–3F).

### 3.4 Effects of exercise training on inflammatory response in SHR

**Fig 4:**
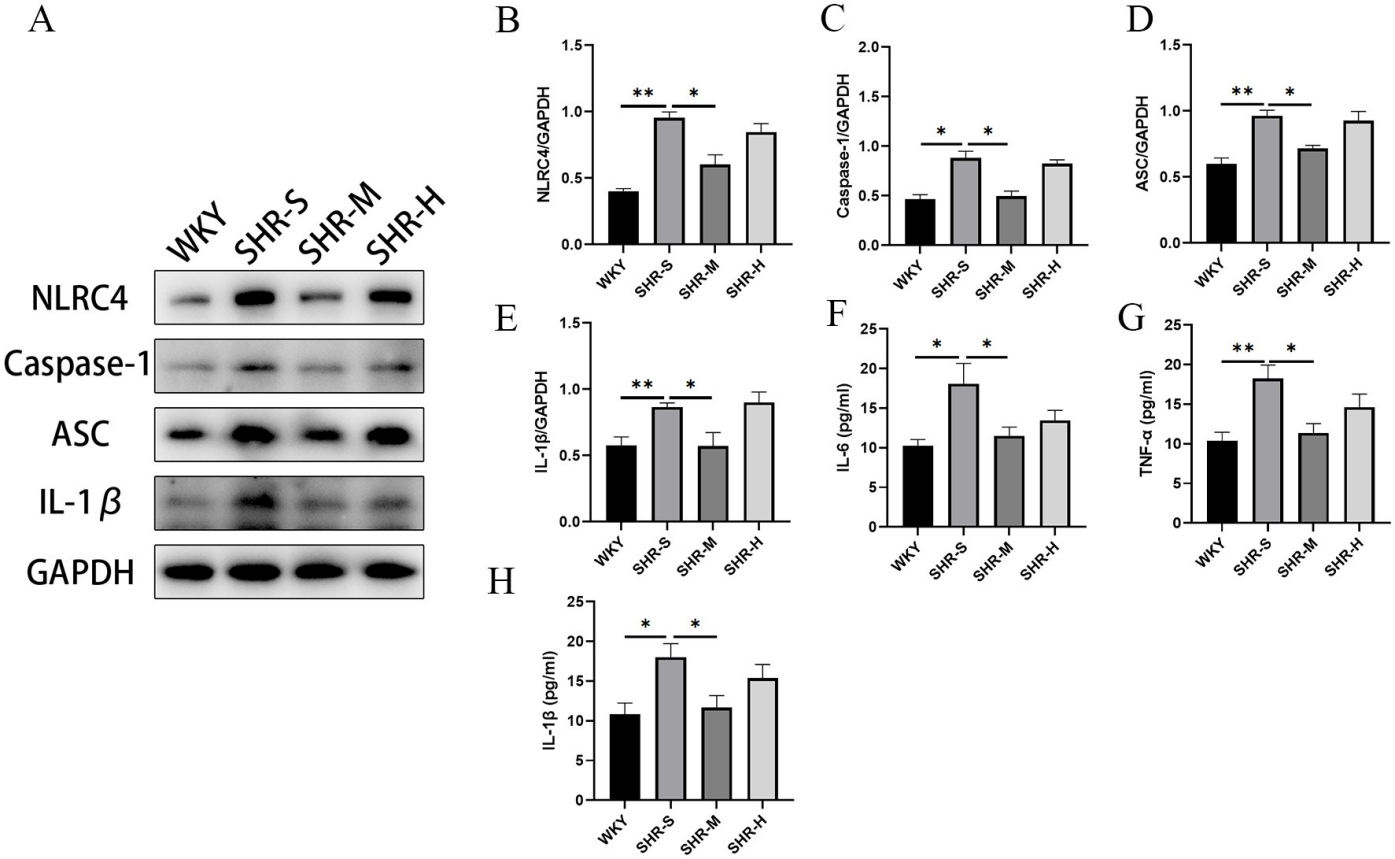
Effects of MICT and HIIT on inflammatory response in SHR. (A) Western blot was used to detect the protein expressions of (B) NLRC4, (C) Caspase-1, (D) ASC and (E) IL-1β in WKY group, SHR-S group, SHR-M group, SHR-H group. ELISA detection of (F) IL-6 and (G) TNF-α (H) IL-1β for evaluating the concentration of pro-inflammatory factors in serum. Data are presented as means ± SEM. N = 3 in each group. *p< 0.05, **p< 0.01.

We detected the expression of NLRC4, Caspase-1, ASC and IL-1β in the kidney to explore whether exercise training affected the activation of NLRC4 inflammasome. We observed the expression levels of NLRC4, Caspase-1, ASC, and IL-1β elevated in the SHR-S group, which were decreased in the SHR-M group. Whereas HIIT did not decrease the expression levels of NLRC4, Caspase-1, ASC, and IL-1β (Fig 4A–4E).

To investigate whether exercise training could alleviate inflammation levels, we used enzyme-linked immunosorbent assay (ELISA) kits to measure the concentrations of pro-inflammatory cytokines IL-1β, IL-6 and TNF-α in serum. Compared with WKY group, the expression levels of IL-1β, IL-6 and TNF-α were significantly higher in the SHR-S group. Moreover, SHR-M group had significantly lower IL-1β, IL-6 and TNF-α levels than the SHR-S group. However, HIIT did not decrease the expression levels of IL-1β, IL-6 and TNF-α (Fig 4F–4H).

### 3.5 Effects of exercise training on TLR4, p-p65 and p-IκBα expression

**Fig 5:**
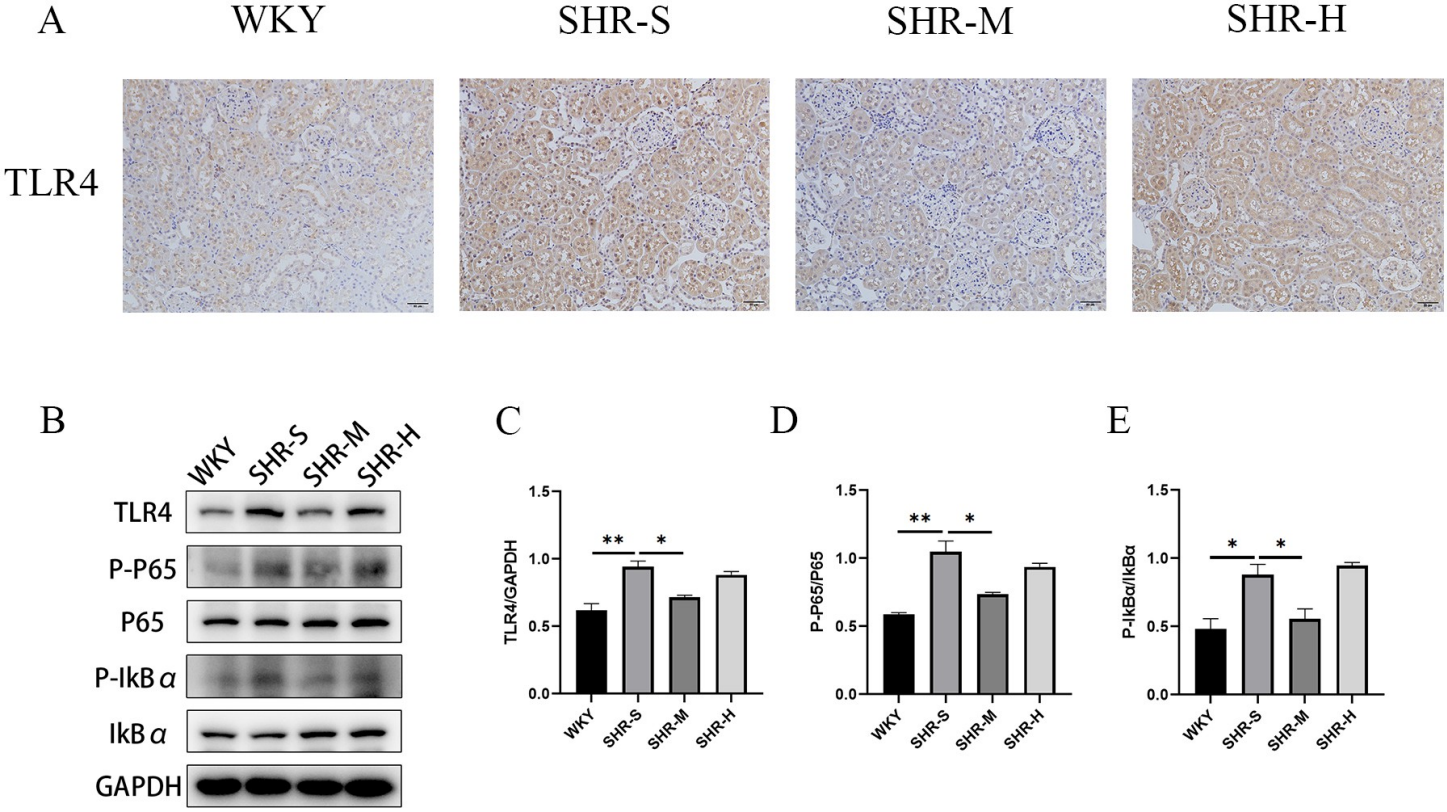
Effects of MICT and HIIT on TLR4/NF-κB pathway in SHR. (A) Immunohistochemical staining result of TLR4 in the kidney tissue. The brown area is positive for expression of TLR4. (B) Western blot was used to detect the expression levels of (C)TLR4, (D)p-p65 and p65, (E) p-IκBα and IκBα in WKY group, SHR-S group, SHR-M group, SHR-H group. Data are presented as means ± SEM. N = 3 in each group. *p< 0.05, **p< 0.01.

We investigated whether the improvement effects of exercise training on renal fibrosis was related to the TLR4 signaling pathway considering the crucial role of the TLR4/NF-κB pathway in inflammatory response. The immunohistochemistry staining was performed to determine the expression of TLR4. The results showed that MICT significantly decreased the expression of TLR4 in SHR (Fig 5A). The expression levels of TLR4, NF-κB p65 and IκBα in renal tissues were detected by western blot. The expression level of TLR4, p-p65/p65 ratio and p-IκBα/IκBα ratio were significantly increased in SHR-S group compared to WKY group. In addition, MICT markedly inhibited the activation of the TLR4/NF-κB pathway by down-regulating the expression level of TLR4, p-p65/p65 ratio and p-IκBα/IκBα ratio. However, we found no significant differences between SHR-S group and SHR-H group.

### 3.6 TAK-242 blocked the activation of TLR4/NF-κB pathway in Ang II-induced HK-2 cells

**Fig 6:**
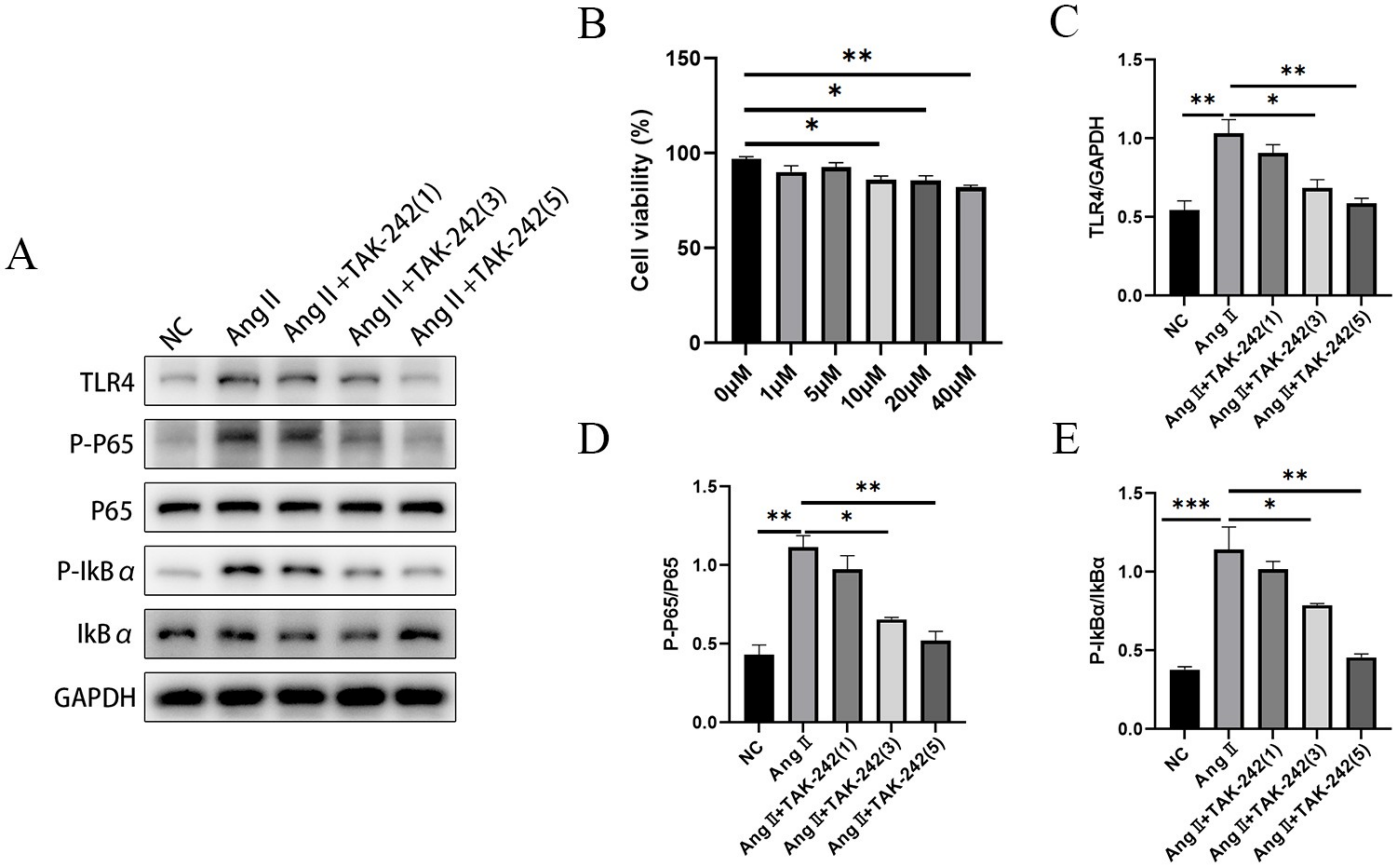
TAK-242 inhibited TLR4/NF-κB pathway in Ang II-induced HK-2 cells. (B) CCK-8 assay was used to determine the cell viability. We found that 10 μM Ang II significantly reduced the cell viability of HK-2 cells. We selected 10 μM Ang II for inducing HN model *in vitro* based on the results of CCK-8. HK-2 cells were treated with 1, 3, 5 μM TAK-242 for 2 hours prior to 10 μM Ang II for 24 hours. (A) Western blot was used to detect the protein levels of (C) TLR4, (D) p-p65 and p65, (E) p-IκBα and IκBα. Data are presented as means ± SEM. N = 3 in each group. *p< 0.05, **p< 0.01, ***p< 0.001.

To further explore whether there is a relationship between TLR4 and NLRC4 in HN *in vitro*, we selected the Ang II at a dose of 10 μΜ based on the CCK-8 results (Fig 6B). Firstly, HK-2 cells were pre-treated with different concentrations of TAK-242 (a TLR4 inhibitor) for 2 hours, and then incubated with 10 μΜ Ang II for 24 hours. The expression level of TLR4, p-IκBα/IκBα ratio and p-p65/p65 ratio were observed to be elevated in Ang II-treated HK-2 cells in western blot results (Fig 6A), while these effects were distinctly alleviated in TAK-242 treatment group (3 and 5 μM), suggesting that TAK-242 inactivated the TLR4/NF-κB pathway in Ang II -induced HK-2 cells (Fig 6C–6E).

### 3.7 TLR4 inhibition protected HK-2 cells from Ang II-induced fibrosis and suppressed the activation of NLRC4 inflammasome

**Fig 7:**
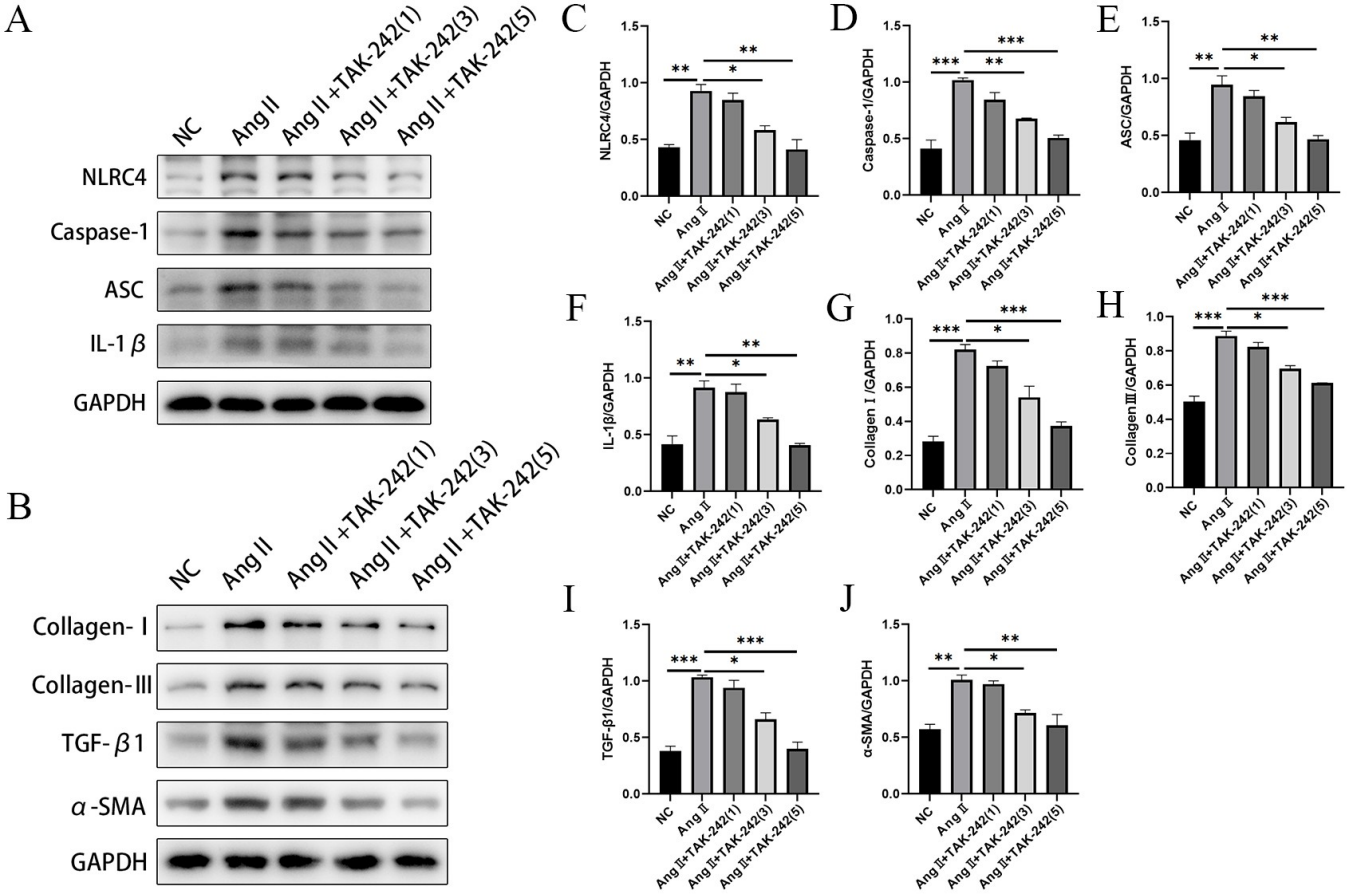
Effects of TAK-242 on fibrosis and activation of NLRC4 inflammasome in Ang II-induced HK-2 cells. HK-2 cells were treated with 1, 3, 5 μM TAK-242 for 2 hours prior to 10 μM Ang II for 24 hours. (A, B) Western blot was used to detect the protein levels of (C-F) fibrosis-related molecules and (G-J) NLRC4 inflammasome. Data are presented as means ± SEM. N = 3 in each group. *p< 0.05, **p< 0.01, ***p< 0.001.

To further investigate the effects of TLR4/NF-κB pathway on fibrosis in HN *in vitro*, the protein levels of collagen I, collagen III, TGF-β1 and α-SMA were detected in the Ang II-induced HK-2 cells by Western blot (Fig 7A). When compared with the control group, the key fibrotic protein levels were significantly increased in the Ang II-induced group. However, TAK-242 treatment at 3 and 5 μM remarkably lowered the expression of fibrosis-related proteins, indicating that TLR4 inhibition could attenuate Ang II-induced fibrosis in HN *in vitro* (Fig 7C–7F). In addition, we performed Western blot to explore whether there is a relationship between TLR4 and NLRC4 (Fig 7B). Western blot results showed that TAK-242 treatment at 3 and 5 μM significantly reduced the expression levels of NLRC4, Caspase-1, ASC, and IL-1β in Ang II-induced HK-2 cells, indicating that inhibiting TLR4 to some extent reduced the activation of NLRC4 inflammasome (Fig 7G–7J).

## 4 Discussion

In the present study, we investigated the renoprotective effects and underlying molecular mechanism of MICT and HIIT on the pathogenesis of HN in SHR. Our research indicated that MICT reduced blood pressure, improved renal function, and alleviated renal pathological damage in SHR. We also found that MICT alleviated renal fibrosis and inflammatory response as well as inhibited TLR4/NF-κB pathway and the activation of NLRC4 inflammasome in SHR. In addition, *in vitro* experiments showed that inhibiting TLR4 could improve Ang II-induced fibrosis in HK-2 cells and down-regulate the activation of NLRC4 inflammasome. Therefore, we identified a relationship between TLR4/NF-κB pathway and the activation of NLRC4 inflammasome.

Hypertensive nephropathy is the second main cause of ESRD after diabetic nephropathy, which seriously do harm to public health [36]. Renal fibrosis is a prevalent pathological process in chronic kidney disease, ultimately leading to renal dysfunction and failure [37, 38]. We found that the expression levels of collagen I, collagen Ш, TGF-β1, α-SMA were decreased in SHR-M group, indicating that MICT alleviated renal fibrosis. TGF-β1 can activate downstream Smad3 signaling, playing a crucial role in fibrosis. Smad3 can interact with other signaling pathways, including the ERK/p38 MAPK and NF-κB pathways to regulate inflammation and fibrosis [39].

Activation of NF-κB pathway triggers a series of inflammatory response, leading to the release of various pro-inflammatory cytokines [40]. Substantial studies have demonstrated that inflammation is involved in the pathogenesis of HN [41–43]. Inflammation and fibrosis interact with each other, contributing to the progressive deterioration of renal function [44]. Mesangial cells (MCs) of kidney stimulated with AngII produce ROS and release IL-1β, TNF-α, the macrophage chemoattractant protein (MCP-1) and TGF-β1 [45, 46]. These inflammatory mediators contribute to fibrosis, reducing renal blood flow and permeability, ultimately leading to decrease in glomerular filtration function [47]. In our study, MICT inhibited the activation of TLR4/NF-κB signaling pathway, and decreased serum concentration of IL-1β, IL-6 and TNF-α. However, these effects were not found in the SHR-H group. We believe that the improvement of renal fibrosis in the SHR-M group is related to the inhibition of the TLR4/NF-κB pathway and the reduction of inflammatory cytokine levels.

NLRP3 inflammasome has been demonstrated to be involved in the pathogenesis of chronic cardiovascular diseases, including hypertension [21, 48, 49]. It is well known that endothelial dysfunction mediated by NLRP3-dependent pyroptosis is one of the main factors in the pathophysiology of hypertension [50]. A study has shown that NLRP3 inhibitor MCC950 reduced inflammation levels and epithelial-mesenchymal transition (EMT) by inhibiting the activation of NLRP3 inflammasome in 1K/DOCA/salt-induced hypertension, which is related to MCC950-sensitive accumulation of M2-like macrophages in the kidney [51]. The NLRP3 inflammasome mediates maturation of caspase-1 and IL-1β to form inflammatory cascade, leading to extracellular matrix (ECM) deposition and fibrosis [52, 53]. Another study has shown that NLRP3 and NLRC4 inflammasomes are activated in macrophages exposed to hypertonic conditions, inducing activation of caspase-1 and IL-1β [54]. However, there is limited research on NLRC4 inflammasome and its effects in fibrosis are not very clear [22]. A previous study has demonstrated that NLRC4 mediated IL-1R antagonist (IL-1Ra) through NF-κB to bind IL-1β, alleviating cystic fibrosis [55]. In addition, it has also been reported that constitutive activation of the NLRC4 inflammasome to facilitate regeneration of liver cells and mitigate liver fibrosis after partial hepatectomy [56]. Whereas we found that the expression levels of NLRC4 inflammasome and fibrosis related proteins were decreased in SHR-M group, indicating MICT reduced renal fibrosis in SHR by inhibiting the activation of NLRC4 inflammasome. *In vitro* HN model of HK-2 cells induced by Ang II, we further observed a decrease in the expression level of NLRC4 inflammasome when TLR4 was inhibited, which seems to be related to the inhibition of TLR4/NF-κB pathway.

Current research indicates that drug therapy combined with aerobic exercise is superior to single drug therapy in cardiovascular disease [57]. Aerobic exercise has been proven to improve endothelial function [58], alleviate inflammation [59] and fibrosis [60]. Furthermore, Exercise has been demonstrated to regulate various signaling pathways in inflammatory diseases, including Sirt1/AMPK/Nrf2 pathway [61], PI3K/Akt/NF-κB pathway [62]. MICT has always been considered as the cornerstone of non-pharmacological therapies for improving cardiovascular diseases such as hypertension [63]. Our previous research has shown that MICT inhibited activation of the TLR4/NF-κB/NLRP3 pathway to improve inflammatory response and oxidative stress in SHR [64]. In recent years, HIIT has been suggested as an alternate treatment to MICT due to its advantage of time efficiency [65]. A systematic review and meta-analysis showed that HIIT is more effective than MICT in improving brachial artery vascular function, which is related to the improvement of inflammation, oxidative stress and insulin sensitivity [66]. In addition, a study showed that HIIT is more effective than MICT in terms of enhancing cardiorespiratory health [67]. But according to another study, HIIT and MICT had comparable effects in lowering resting blood pressure in adults with pre-to established hypertension [68]. Our research indicated that MICT was more effective at improving blood pressure and renal fibrosis than HIIT in SHR, so we recommend that hypertensive patients exercise with 50%-60% VO_2max_.

However, our research still has limitations, we only intervened in TLR4 to observe changes in expression of NLRC4, and did not explore the downstream molecular mechanism of NLRC4 inflammasome induced fibrosis. Future research may focus on the exact molecular mechanism of NLRC4 affecting fibrosis. In addition, further clinical trials are needed to verify whether our exercise prescription is applicable to humans.

## 5 Conclusion

In summary, our present findings suggest that MICT reduced renal damage, inflammatory response, and renal fibrosis to ameliorate HN. TLR4 inhibition has been demonstrated *in vitro* to repress the activation of NLRC4 and alleviate fibrosis in Ang II-induced HK-2 cells. We also found that NLRC4 has the potential to become a new therapeutic target for hypertensive nephropathy. The present study adds more information to better understand the mechanism behind aerobic exercise involved in HN and provides reference for the study of exercise prescriptions for hypertension to some extent. In the future, the research may aim to the downstream molecular mechanism of NLRC4 inflammasome induced fibrosis.

## Supporting information

S1 Fig Body weight, blood pressure, heart rate and renal function of rats. (TIF)

S2 Fig HE staining results of rats. We selected the magnification of 200x to capture images from 6 different fields of view under the microscope.
(TIF)

S3 Fig Masson trichrome staining results of rats. We selected the magnification of 200x to capture images from 6 different fields of view under the microscope.
(TIF)

S4 Fig Concentration of IL-1β, IL-6 and TNF-α (pg/mL) in serum of rats. ELISA was used to detect the concentration of IL-1β, IL-6 and TNF-α (pg/mL) of rats.
(TIF)

S5 Fig Immunohistochemical staining results of rats. We selected the magnification of 200x to capture images from 6 different fields of view under the microscope.
(TIF)
