# Supplementary figures and images for: MICT ameliorates hypertensive nephropathy by inhibiting TLR4/NF-κB pathway and down-regulating NLRC4 inflammasome
Source: PLoS One. 2024 Jul 25;19(7):e0306137. doi: 10.1371/journal.pone.0306137 (PMC11271930; doi:10.1371/journal.pone.0306137)

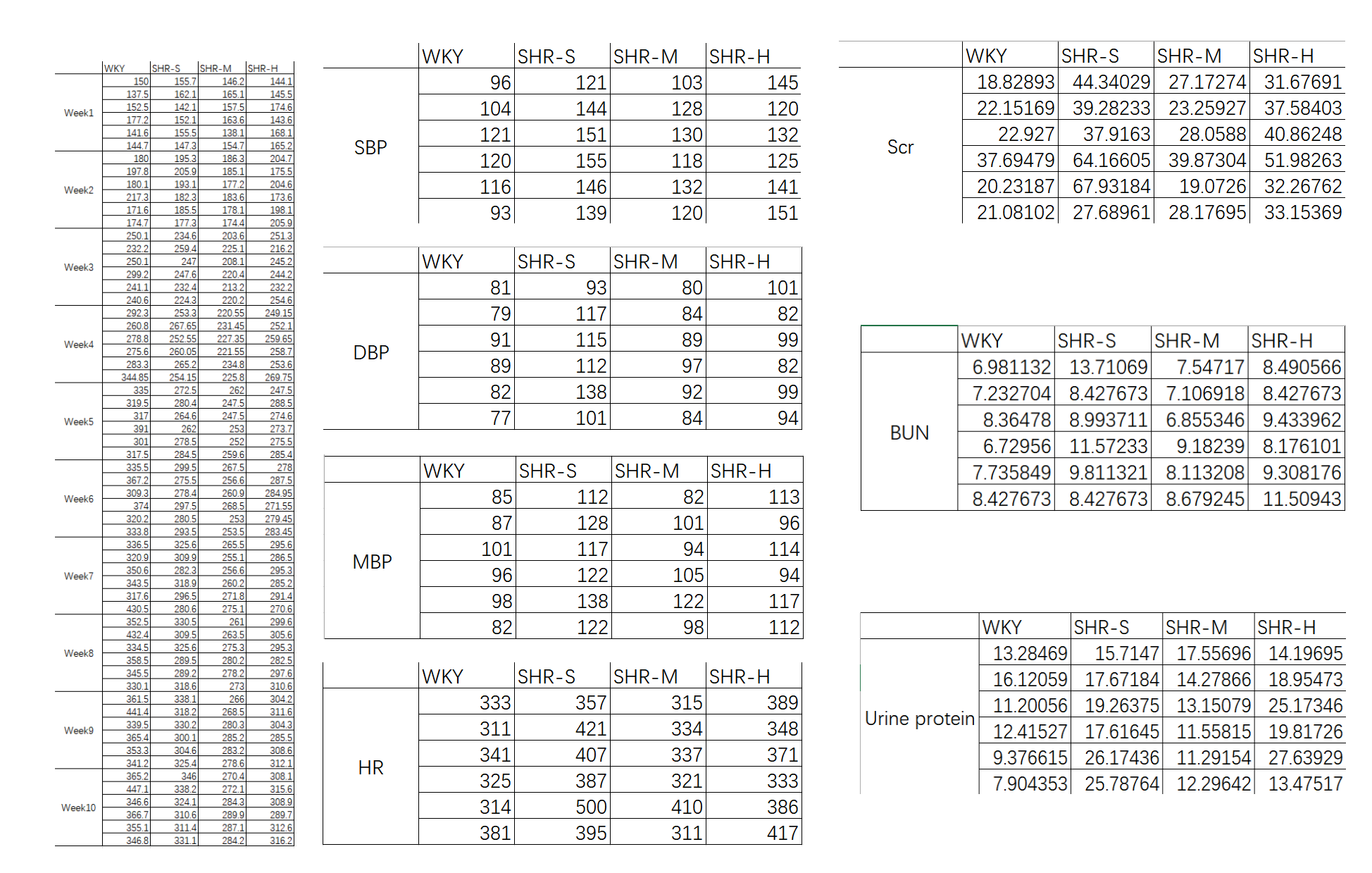

Supplement: S1 Fig — (TIF) [file pone.0306137.s001.tif]

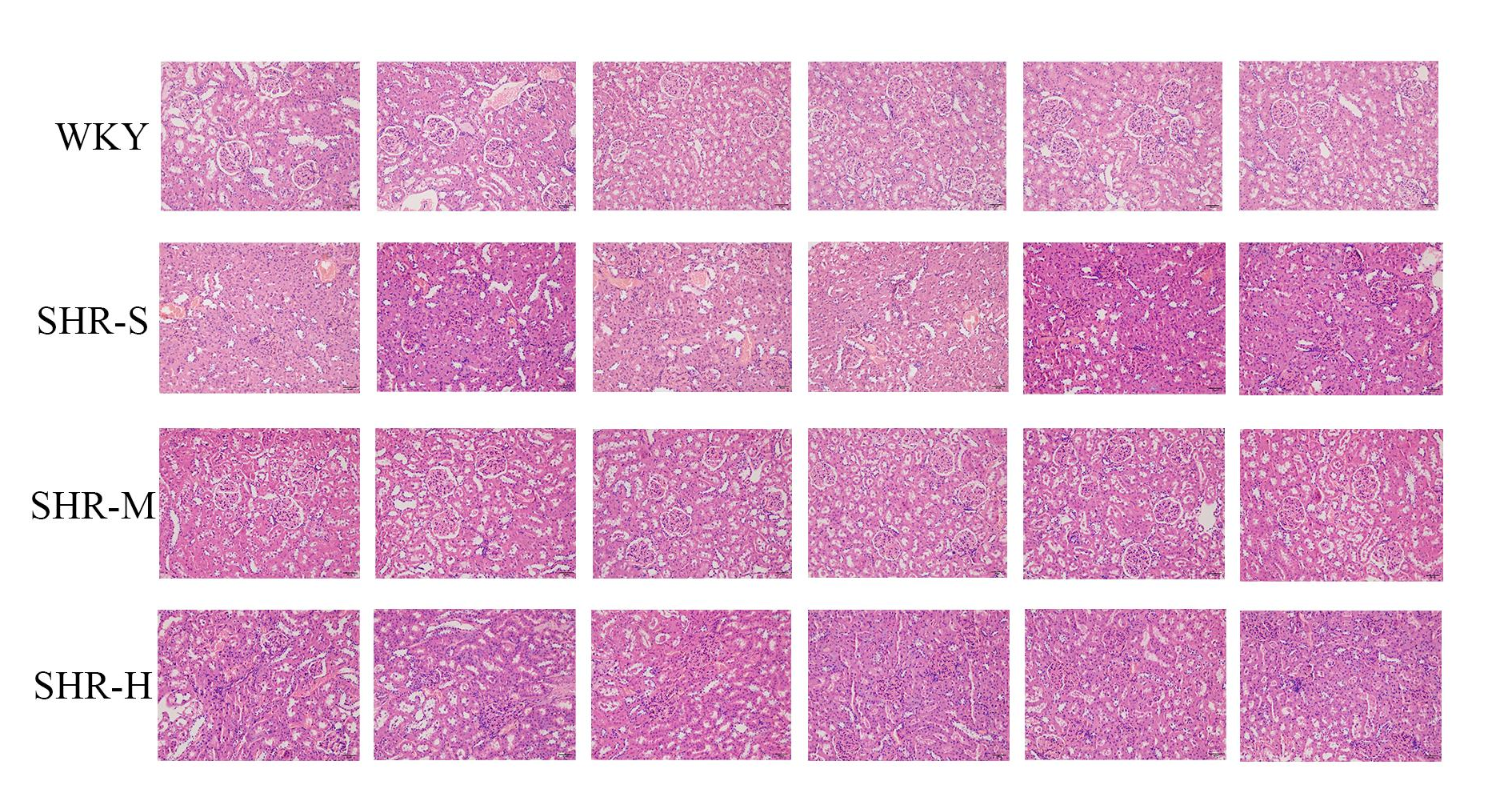

Supplement: S2 Fig — We selected the magnification of 200x to capture images from 6 different fields of view under the microscope. (TIF) [file pone.0306137.s002.tif]

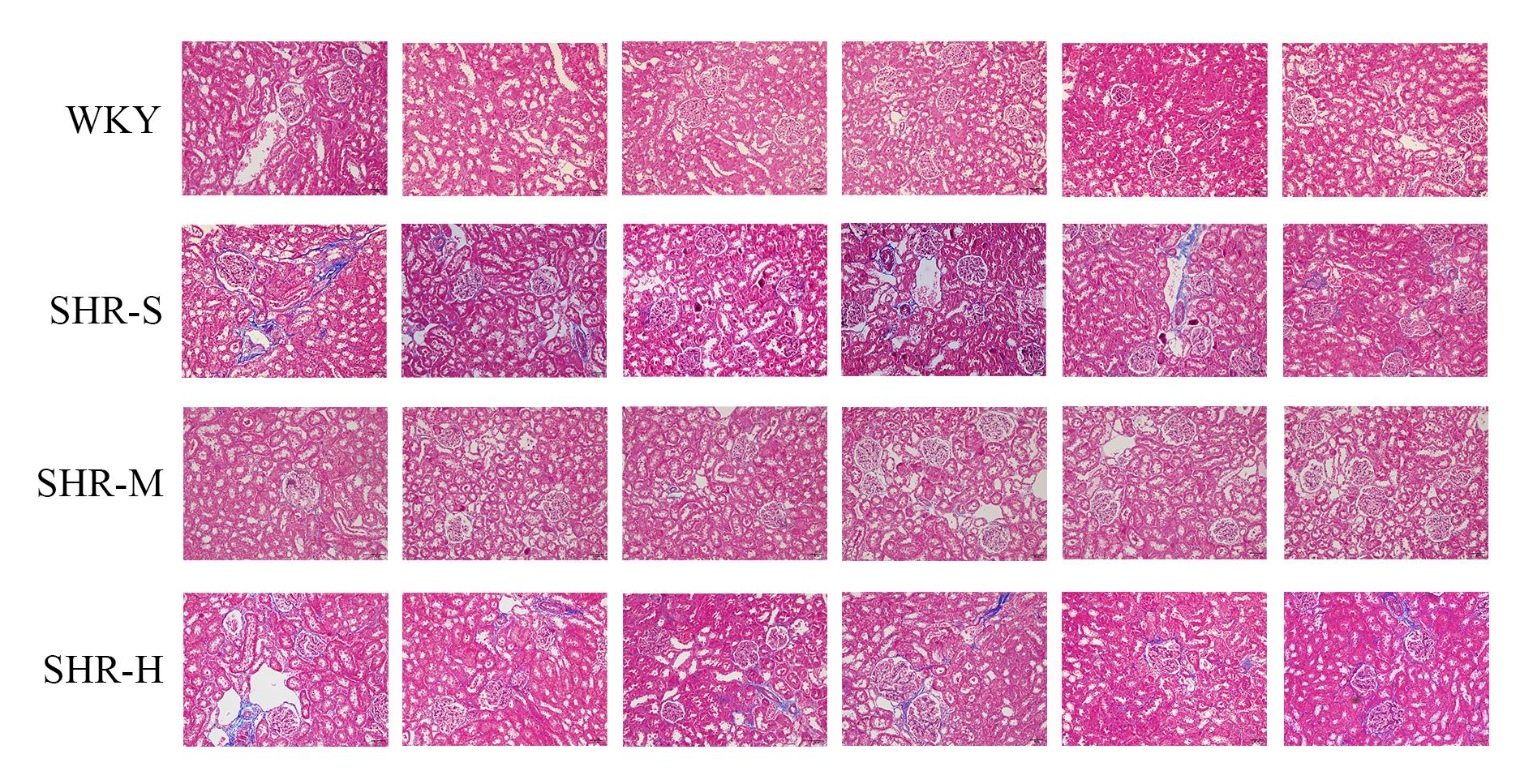

Supplement: S3 Fig — We selected the magnification of 200x to capture images from 6 different fields of view under the microscope. (TIF) [file pone.0306137.s003.tif]

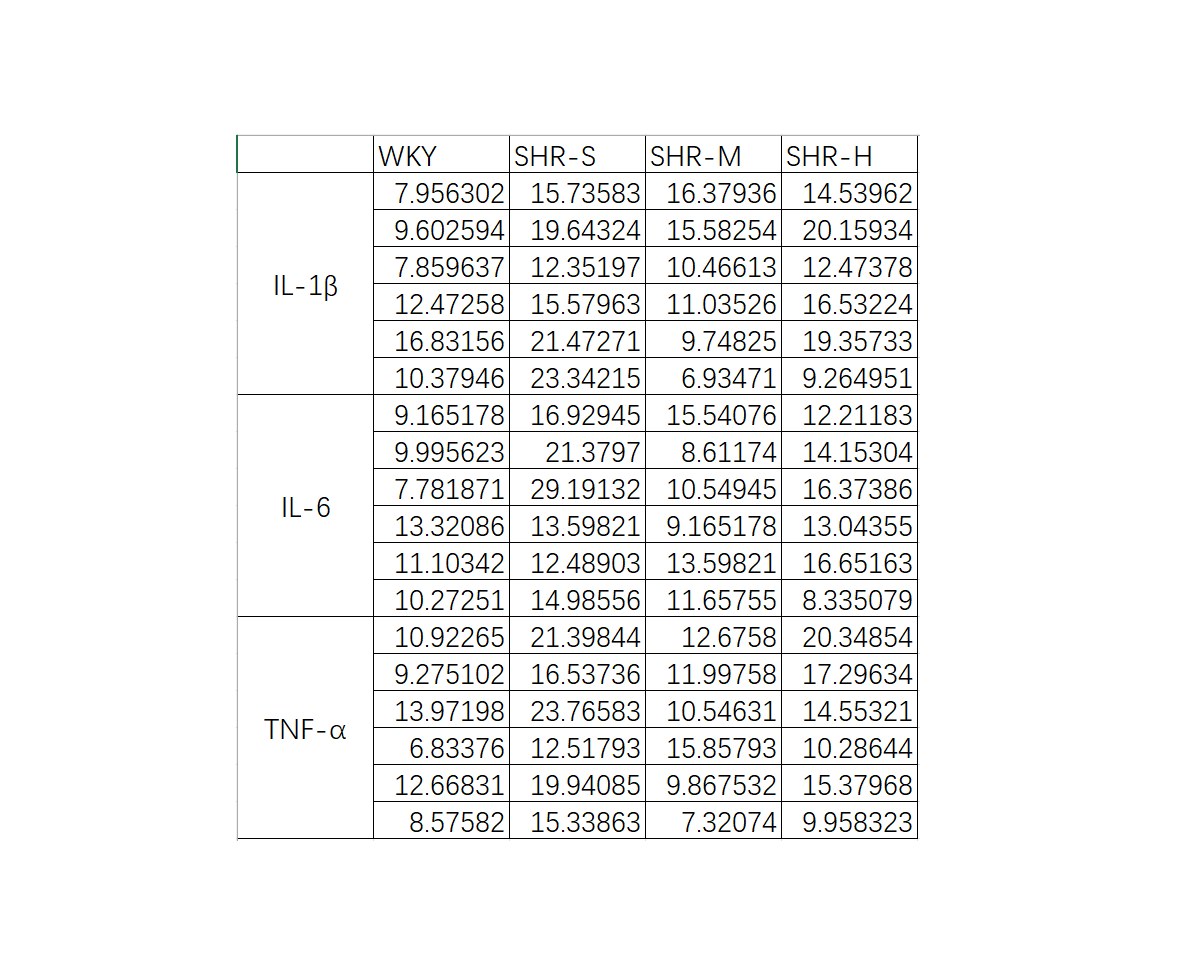

Supplement: S4 Fig — ELISA was used to detect the concentration of IL-1β, IL-6 and TNF-α (pg/mL) of rats. (TIF) [file pone.0306137.s004.tif]

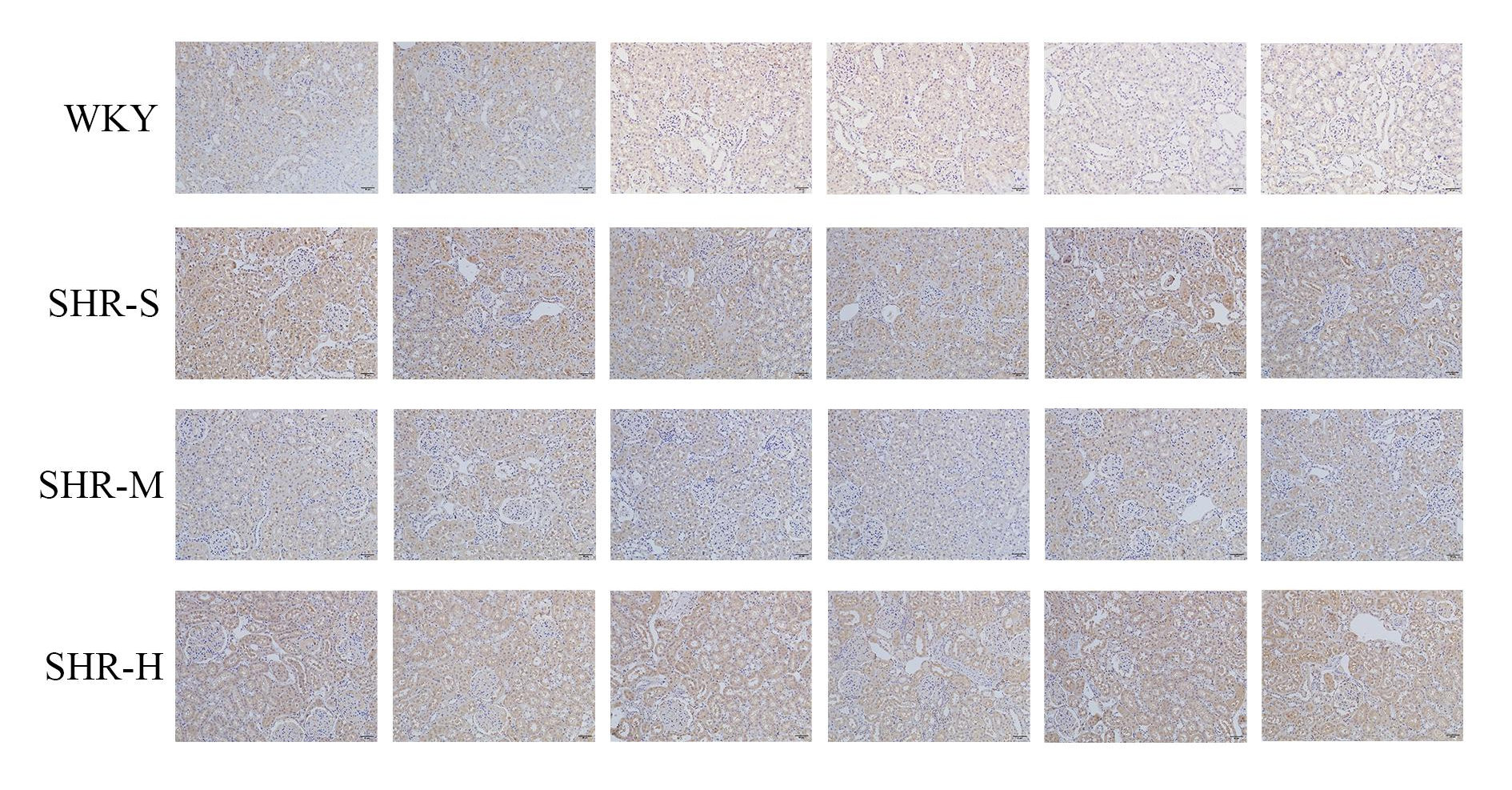

Supplement: S5 Fig — We selected the magnification of 200x to capture images from 6 different fields of view under the microscope. (TIF) [file pone.0306137.s005.tif]
